# Association Between Carbohydrate Quality Index During Pregnancy and Risk for Large-for-Gestational-Age Neonates: Results from the BORN 2020 Study

**DOI:** 10.3390/children12070955

**Published:** 2025-07-20

**Authors:** Antigoni Tranidou, Antonios Siargkas, Ioannis Tsakiridis, Emmanouela Magriplis, Aikaterini Apostolopoulou, Michail Chourdakis, Themistoklis Dagklis

**Affiliations:** 1 3rd Department of Obstetrics and Gynecology, School of Medicine, Faculty of Health Sciences, Aristotle University of Thessaloniki, 541 24 Thessaloniki, Greece; antigoni.tranidou@gmail.com (A.T.); igtsakir@auth.gr (I.T.); 2 Laboratory of Hygiene, Social & Preventive Medicine and Medical Statistics, School of Medicine, Faculty of Health Sciences, Aristotle University of Thessaloniki, 541 24 Thessaloniki, Greece; katapost@yahoo.gr (A.A.); mhourd@auth.gr (M.C.); 3 Department of Food Science and Human Nutrition, Agricultural University of Athens, Iera Oos 75, 118 55 Athens, Greece; emagriplis@eatsmart.gr

**Keywords:** Carbohydrate Quality Index, CQI, pregnancy, nutrition, large for gestational age, LGA, gestational diabetes mellitus, GDM

## Abstract

**Background/Objectives**: To assess the association between early pregnancy carbohydrate quality, as measured by the Carbohydrate Quality Index (CQI), and the risk of delivering a large-for-gestational-age (LGA) infant in a Mediterranean pregnant cohort of northern Greece. **Methods**: We analyzed singleton pregnancies from the BORN 2020 prospective cohort in Greece. Dietary intake was assessed via a validated food frequency questionnaire, and CQI was computed from glycemic index, fiber density, whole-to-refined grain ratio, and solid-to-liquid carbohydrate ratio. Multivariable logistic regression was used to estimate the association between CQI (in tertiles) and LGA risk, defined as birthweight >90th percentile. **Results**: Among the 797 participants, 152 (19.1%) delivered LGA infants, and 117 (14.7%) were diagnosed with GDM. Of those with GDM, 23 (19.7%) delivered LGA infants. In the total population, higher maternal weight (*p* < 0.001), height (*p* = 0.006), and pre-pregnancy BMI (*p* = 0.004) were significantly associated with LGA. A greater proportion of women with LGA had a BMI > 25 (*p* = 0.007). In the GDM subgroup, maternal height remained significantly higher in those who delivered LGA infants (*p* = 0.017). In multivariable models, moderate CQI was consistently associated with increased odds of LGA across all models (Model 1: aOR = 1.60 (95% CI: 1.03–2.50), *p* = 0.037, Model 2: aOR = 1.57 (95% CI: 1.01–2.46), *p* = 0.046, Model 3: aOR = 1.58 (95% CI: 1.01–2.47), *p* = 0.044, Model 4 aOR: 1.70; 95% CI: 1.08–2.72; *p* = 0.023), whereas high CQI was not. In the GDM subgroup, a significant association between high CQI and increased LGA risk was observed in less adjusted models (Model 1 aOR: 6.74; 95% CI: 1.32–56.66; *p* = 0.039, Model 2 aOR: 6.64; 95% CI: 1.27–57.48; *p* = 0.044), but this was attenuated and became non-significant in the fully adjusted model (aOR: 3.05; 95% CI: 0.47–30.22; *p* = 0.28). When examining CQI components individually, no consistent associations were observed. Notably, a higher intake of low-quality carbohydrates (≥50% of energy intake) was significantly associated with increased LGA risk in the total population (aOR: 4.25; 95% CI: 1.53–11.67; *p* = 0.005). **Conclusions**: Higher early pregnancy intake of low-quality carbohydrates was associated with an elevated risk of LGA in the general population. However, CQI itself showed a non-linear and inconsistent relationship with LGA, with moderate, but not high, CQI linked to increased risk, particularly in GDM pregnancies, where associations were lost after adjustment. Both carbohydrate quality and quantity evaluations are essential, particularly in high-risk groups, to inform dietary guidance in pregnancy.

## 1. Introduction

The global rise of large-for-gestational-age (LGA) neonates, defined as birthweight above the 90th percentile for gestational age, has critical implications for both perinatal care and long-term metabolic health [1]. LGA neonates face increased risks of delivery complications and are more likely to develop childhood obesity, insulin resistance, and other cardiometabolic disorders, as well as LGA offspring themselves [2]. While LGA arises from a range of maternal and fetal factors, including high maternal body mass index (BMI), disturbances in maternal glucose regulation are increasingly recognized as key contributors [3]. Other important contributors include maternal physical activity, socioeconomic status, and genetic predisposition, all of which may also confound associations between diet and fetal growth [4,5,6]. Moreover, the Hyperglycemia and Adverse Pregnancy Outcomes (HAPO) study demonstrated a continuous association between maternal glucose levels, even below the diagnostic threshold for gestational diabetes mellitus (GDM), and the risk of LGA. This association was independent of BMI and gestational weight gain [7].

Emerging evidence suggests that conditions in the intrauterine environment can have lasting effects on health across the lifespan [8,9]. According to the developmental origins of health and disease hypothesis, disruptions in fetal growth may predispose individuals to chronic conditions in adulthood, including metabolic and cardiovascular diseases [10]. Conversely, supporting appropriate fetal development may contribute to better long-term outcomes [11].

Maternal diet is a highly modifiable factor with a strong influence on fetal growth and development [12,13]. While carbohydrate quantity has been extensively studied in relation to GDM and fetal outcomes [14], increasing attention has shifted toward carbohydrate quality [15]. To date, there are conflicting published data on the effect of diets with a low glycemic index (GI) on the risk of delivering LGA infants. The underlying mechanism may involve enhanced postprandial glucose regulation and reduced fetal hyperinsulinemia [16,17]. Moreover, in pregnancies complicated by GDM, the relationship appears more complex; some evidence suggests that diets rich in high-quality carbohydrates, such as whole grains, legumes, and foods with low GI, may contribute to better glycemic control and more optimal fetal growth patterns, especially in pregnancies complicated by GDM [18]. Nonetheless, other findings reported no significant reduction in LGA incidence among women with GDM [19], suggesting that the interplay between dietary components and fetal growth in GDM is influenced by multiple confounding and modifying factors. Additionally, adherence to a low-GI or low-glycemic-load (GL) diet has been linked to reduced rates of excessive gestational weight gain in women at high risk for GDM [20].

To capture the multidimensional nature of carbohydrate quality, recent nutritional epidemiology has employed composite indices. One such metric is the Carbohydrate Quality Index (CQI) that reflects the overall quality of carbohydrate intake by combining four key components: dietary glycemic index (GI), fiber density, whole-to-refined grain ratio, and the proportion of solid versus liquid carbohydrates, in a single score [21]. In non-pregnant populations, higher CQI scores may be inversely associated with risk of type 2 diabetes and cardiovascular disease [22]. Although CQI has also been applied in pregnancy, particularly in studies investigating GDM, knowledge of its relationship to fetal growth and LGA remains limited, and formal validation in this specific context is still lacking [23]. Given the complex metabolic adaptations of pregnancy and the limitations of single-nutrient markers, a composite index such as CQI may offer a more comprehensive tool for assessing carbohydrate-related risk. Το our knowledge, no study has evaluated the composite CQI in association with LGA in a Mediterranean population. In Mediterranean populations, carbohydrate intake is often rich in grains, legumes, fruits, and vegetables, making CQI evaluation particularly relevant in this dietary context.

We therefore conducted a prospective analysis within the BORN 2020 cohort [24] to examine the association between maternal early-pregnancy carbohydrate quality, quantified by CQI, and the risk of delivering an LGA infant. We also explored carbohydrate quantity in interaction with quality and specifically assessed LGA risk in pregnancies complicated by GDM. We hypothesized that higher maternal CQI, reflecting superior carbohydrate quality, would be independently associated with reduced odds of LGA, even after adjustment for total energy intake, pre-pregnancy BMI, gestational weight gain, and glycemic status. Addressing this gap may inform dietary guidelines aiming to optimize fetal growth trajectories and long-term metabolic health.

## 2. Materials and Methods

### 2.1. Study Design and Population

This analysis is part of the BORN 2020 study, a longitudinal prospective study in Greece. Participants enrolled between July 2020 and December 2022 at the 3rd Department of Obstetrics and Gynecology, School of Medicine, Faculty of Health Sciences, Aristotle University of Thessaloniki, Greece. Written informed consent was obtained from all participants. The Bioethics Committee of Aristotle University of Thessaloniki (protocol code 6.231/Date of approval 29 July 2020) approved the study.

Recruitment took place during the participants’ first routine perinatal visit, which occurred between 11^+0^ and 13^+6^ weeks of gestation. Women were eligible if they were at least 18 years old, carrying a singleton pregnancy, and had no history of diabetes. Exclusion criteria included multiple pregnancies, major health conditions (such as hypertension or renal disorders), adherence to restrictive diets (such as vegetarianism), and any missing or incomplete data. Data on maternal age, pre-pregnancy BMI, parity, smoking status during pregnancy, nutritional data, gestational weight gain, and GDM status (based on a 75 g oral glucose tolerance test at 24–28 weeks) were collected via questionnaire and clinical records.

### 2.2. Dietary Assessment and Carbohydrate Quality Index

At baseline, dietary intake was assessed using a locally validated food frequency questionnaire (FFQ) [25]. Nutrient intake was computed using NutriSurvey software (EBISpro, Willstätt, Germany, version 2007). The Carbohydrate Quality Index (CQI) was computed by summing quintile-based scores (1–5) across four dimensions: glycemic index (GI), fiber density (g/1000 kcal), whole-to-refined grain ratio, and solid-to-liquid carbohydrate ratio (i.e., ratio of carbohydrate intake from solid foods to that from beverages). The composite CQI ranged from 4 (lowest quality) to 20 (highest quality), analyzed both continuously and in quartiles. Carbohydrate intake was also expressed as percentage of total energy (≤40%, 40–50%, ≥50%). CQI was analyzed both as a continuous variable and in cohort-specific quartiles.

### 2.3. GDM Diagnosis

GDM was diagnosed according to the guidelines of the Hellenic Society of Obstetricians and Gynecologists, which are consistent with the criteria set by the HAPO study [7]. In brief, all participants underwent a 75 g oral glucose tolerance test (OGTT) between 24 and 28 weeks of pregnancy. Blood glucose levels were measured at three points: fasting, 1 h, and 2 h post-glucose intake. A GDM diagnosis was made if any of the following thresholds were met or exceeded: 92 mg/dL (fasting), 180 mg/dL (1 h), or 153 mg/dL (2 h). To maintain consistency in the diagnostic timeframe, women who had undergone an OGTT prior to 24 weeks due to early risk factors, and had been diagnosed with GDM, were excluded from the study.

### 2.4. Outcome Ascertainment

Birth weight and gestational age at delivery were abstracted from medical records. LGA was defined as birth weight >90th percentile for gestational age based on Greek birthweight reference centile charts [26].

### 2.5. Statistical Analysis

Regarding the population characteristics, for variables following a normal distribution, mean and SD are provided; otherwise, the median and quartiles are reported. The Shapiro–Wilk test (<50 samples) or Kolmogorov–Smirnov test (≥50 samples) was used to assess normality. The *p*-values were obtained using a t-test for normally distributed variables, Mann–Whitney test for non-normally distributed variables, and chi-squared test for categorical variables. Fisher’s exact test (*n* < 5) or chi-squared test (*n* ≥ 5) was applied for binary variables, contingent on the sample size.

The association between CQI and LGA was examined using multivariable logistic regression models with increasing levels of adjustment: Model 1: energy intake, maternal age, pre-pregnancy BMI, parity, physical activity, gestational weight gain, thyroid disease, supplementation intake, and sedentary time; Model 2: Model 1 plus smoking status; Model 3: Model 2 plus alcohol intake; Model 4: Model 3 plus Mediterranean diet adherence (Trichopoulou score).

Covariates were selected based on their established or potential role as determinants of fetal growth and confounders of maternal diet–birthweight associations. We applied models with increasing levels of covariate adjustment to evaluate whether the association between CQI and LGA remained consistent after accounting for maternal, behavioral, and dietary factors. Subgroup analyses were also conducted among women with GDM using the same modeling structure.

Analyses were performed on (i) the entire population and (ii) the subset of participants with GDM. Additional models explored associations between individual CQI components (GI, fiber, grain ratios, carbohydrate ratios) and LGA risk. Interactions between carbohydrate quality and quantity were also assessed in relation to LGA.

## 3. Results

**Figure 1:**
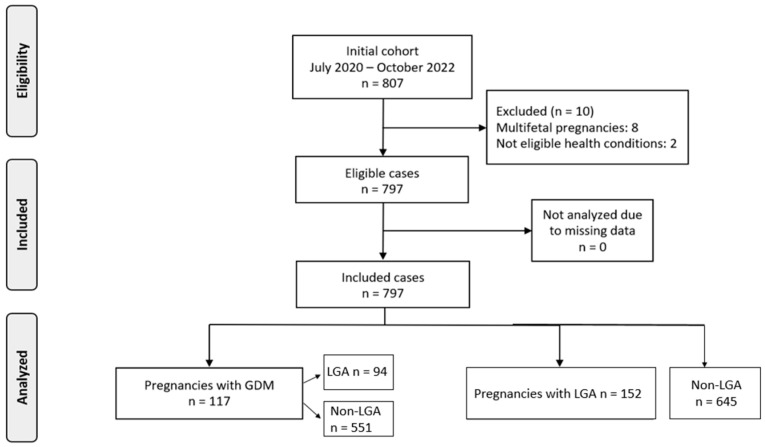
Flowchart of population recruitment.

A total of 807 pregnancies were initially assessed for participation in the study. Following the exclusion of 10 cases, due to either multifetal gestation (*n* = 8) or health-related criteria (*n* = 2), 797 pregnancies met the study’s eligibility requirements and were included in the final analysis. Among these, 117 women (14.7%) were diagnosed with gestational diabetes mellitus (GDM), while the remaining 680 did not develop GDM (Figure 1).

To better understand the relationship between GDM and fetal growth, the sample was further categorized based on birthweight outcomes. Specifically, 152 neonates (19.1%) were classified as LGA, while 645 (80.9%) fell within the normal weight range. Within the GDM subgroup, 23 women gave birth to LGA and 94 to non-LGA infants. Among those without GDM, 129 delivered LGA infants and 551 delivered non-LGA infants.

Table 1 presents the maternal characteristics stratified by LGA status and by GDM subgroups. In the overall population, women who delivered LGA infants had significantly higher pre-pregnancy weight (66 kg vs. 63 kg, *p* < 0.001), height (167 cm vs. 165 cm, *p* = 0.006), and BMI (23.8 vs. 22.7 kg/m^2^, *p* = 0.004) compared to those with non-LGA births. Additionally, a greater proportion of women in the LGA group had a BMI over 25 (42.1% vs. 30.4%, *p* = 0.007). However, maternal age and the proportion of women older than 35 years did not differ significantly between LGA and non-LGA groups (*p* = 0.98 and *p* = 0.95, respectively) nor did rates of smoking, assisted reproduction, thyroid disease, or GDM. Within the subgroup of women diagnosed with GDM, those who delivered LGA infants were significantly taller than those who did not (168.2 cm vs. 164.7 cm, *p* = 0.017), but no significant differences were found in weight, BMI, age, or comorbidities.

As presented in Table 2, the association between the CQI and the risk of LGA infants was evaluated across the total population and in women with GDM, using four adjusted models. In **Model 1**, we observed that moderate carbohydrate quality (CQI) was associated with an increased risk of LGA (aOR: 1.6, 95% CI: 1.03, 2.5, *p* = 0.037). However, the association was not statistically significant in the high-CQI group (aOR: 1.29, 95% CI: 0.81, 2.07, *p* = 0.28). The *p*-value for trend across CQI groups was 0.23, indicating no clear linear trend. In **Model 2**, the findings were similar, with moderate CQI remaining statistically significant (aOR: 1.57, 95% CI: 1.01, 2.46, *p* = 0.046), and no significant association for the high-CQI group. The *p*-value for trend was 0.28. In **Model 3**, moderate CQI again showed a significant association with LGA risk (aOR: 1.58, 95% CI: 1.01, 2.47, *p* = 0.044), while the high-CQI group remained non-significant. The *p*-value for trend was 0.33. In **Model 4**, the association for moderate CQI persisted (aOR: 1.7, 95% CI: 1.08, 2.72, *p* = 0.023), with no significant association for high-CQI (aOR: 1.44, 95% CI: 0.86, 2.4, *p* = 0.16). The *p*-value for trend was 0.12, suggesting no clear linear relationship between CQI and LGA risk.

In women with GDM, the results showed a stronger association between carbohydrate quality and the risk of LGA. In **Model 1**, women in the high-CQI group had a significantly increased risk of delivering LGA infants (aOR: 6.74, 95% CI: 1.32, 56.66, *p* = 0.039). The moderate CQI group also showed a trend toward increased risk (aOR: 3.85, 95% CI: 0.72, 31.93, *p* = 0.15). The *p*-value for the trend was 0.041, indicating a statistically significant trend in favor of higher CQI leading to higher risk of LGA in this subgroup. In **Model 2**, the association for high CQI remained significant (aOR: 6.64, 95% CI: 1.27, 57.48, *p* = 0.044), and the moderate-CQI group continued to show an increased risk (aOR: 3.81, 95% CI: 0.71, 32.02, *p* = 0.15). The *p*-value for the trend was 0.045, further supporting the trend in this subgroup. In **Model 3**, the association remained strong for the high-CQI group (aOR: 6.27, 95% CI: 1.2, 54.33, *p* = 0.051), although the *p*-value was just above 0.05. The *p*-value for the trend was 0.052, which was marginally non-significant. Finally, in **Model 4**, while the odds ratios decreased, the association remained non-significant for both moderate- and high-CQI groups (aOR: 1.9, 95% CI: 0.28, 18.14, *p* = 0.53 for moderate; aOR: 3.05, 95% CI: 0.47, 30.22, *p* = 0.28 for high), and the *p*-value for the trend was 0.3, indicating no significant trend in the most fully adjusted model. The initial association observed in the less adjusted models may have been confounded by other maternal or lifestyle factors. It is also important to note that the GDM subgroup was relatively small, limiting the statistical power of these analyses.

As presented in Table 3, the adjusted associations between various components of the Carbohydrate Quality Index (CQI) and the risk of large-for-gestational-age (LGA) infants were assessed across the total population and in women with gestational diabetes mellitus (GDM). These components include glycemic index (GI), dietary fiber, solid-to-total carbohydrate ratio, and whole-to-total grain ratio.

For GI, no significant association was found with LGA risk in the total population or the GDM subgroup. In the total population, women in the medium-GI group (T2) had an aOR of 0.95 (95% CI: 0.61, 1.5, *p* = 0.85), and women in the high-GI group (T3) had an aOR of 0.79 (95% CI: 0.49, 1.28, *p* = 0.36). Similarly, in the GDM subgroup, no significant association was observed, with aORs of 0.76 (95% CI: 0.19, 2.76, *p* = 0.68) for the medium-GI group and 0.45 (95% CI: 0.07, 2.15, *p* = 0.34) for the high-GI group. These findings suggest that GI does not significantly impact LGA risk in either the total population or the GDM subgroup.

For dietary fiber, no significant association was observed with LGA risk in the total population. Women in the medium-fiber group (T2) had an aOR of 1.38 (95% CI: 0.87, 2.22, *p* = 0.17), and those in the high-fiber group (T3) had an aOR of 1.25 (95% CI: 0.71, 2.2, *p* = 0.43). However, in the GDM subgroup, there was a trend towards an increased risk of LGA with higher dietary fiber intake. In the high-fiber group (T3), the aOR was 3.15 (95% CI: 0.59, 20.6, *p* = 0.19), although this result did not reach statistical significance. This suggests that, while dietary fiber intake does not have a significant impact on LGA risk in the total population, it may play a role in the GDM subgroup, albeit with marginal significance.

Regarding the solid-to-total carbohydrate ratio, no significant association with LGA risk was found in either the total population or the GDM subgroup. In the total population, the aOR for the medium group (T2) was 0.98 (95% CI: 0.62, 1.55, *p* = 0.96), and for the high group (T3), the aOR was 1.24 (95% CI: 0.79, 1.95, *p* = 0.34). In the GDM subgroup, the medium group (T2) had an aOR of 0.55 (95% CI: 0.13, 2.16, *p* = 0.4), and the high group (T3) had an aOR of 0.66 (95% CI: 0.16, 2.48, *p* = 0.55). These findings suggest that the solid-to-total carbohydrate ratio does not have a significant effect on LGA risk in either group.

For the whole-to-total grain ratio, no significant associations were found with LGA risk in either the total population or the GDM subgroup. In the total population, the aOR for the medium group (T2) was 1.31 (95% CI: 0.83, 2.03, *p* = 0.23), and for the high group (T3), the aOR was 1.21 (95% CI: 0.76, 1.91, *p* = 0.41). In the GDM subgroup, the aORs were 2.3 (95% CI: 0.49, 12.28, *p* = 0.3) for the medium group and 2.29 (95% CI: 0.55, 11.38, *p* = 0.27) for the high group, both of which were not statistically significant.

As shown in Table 4, the relationship between carbohydrate quality and quantity in relation to the risk of LGA infants was analyzed across the total population and within the subgroup of women with GDM.

In the total population analysis, carbohydrate quality was assessed across three intake categories: low, moderate, and high carbohydrate quality. For the low carbohydrate quality group, there were no significant associations between carbohydrate intake and LGA risk in any of the intake categories. Specifically, for carbohydrate intake of 40–50% of energy (E), the aOR for LGA was 1.78 (95% CI: 0.83, 3.82, *p* = 0.14) for the moderate group and 0.87 (95% CI: 0.4, 1.88, *p* = 0.73) for the high group. For carbohydrate intake of ≥50% E, the risk of LGA was significantly increased in the low carbohydrate quality group, with an aOR of 4.25 (95% CI: 1.53, 11.67, *p* = 0.005), indicating that a higher carbohydrate intake with low quality was associated with a higher risk of LGA. In contrast, no significant associations were observed in the moderate or high carbohydrate quality groups for ≥50% E intake, with aORs of 1.63 (95% CI: 0.69, 3.81, *p* = 0.25) and 0.53 (95% CI: 0.16, 1.52, *p* = 0.26), respectively.

In the GDM subgroup, carbohydrate intake and its association with LGA risk were more variable. For 40–50% E intake, the analysis of the low carbohydrate quality group showed a significant association, with an aOR of 6.14× 10^−10^ (*p* = 0.1), though the confidence interval could not be determined due to insufficient data. In the high carbohydrate quality group, the association was not significant (aOR: 1.12, 95% CI: 0.11, 10.47, *p* = 0.92). For carbohydrate intake of ≥50% E, the low carbohydrate quality group showed a very low aOR of 0.01 (*p* = 0.1), suggesting little or no impact of high carbohydrate intake on LGA risk. In the high carbohydrate quality group, the aOR was 4.05 (95% CI: 0.13, 165.61, *p* = 0.43), with a very wide confidence interval and no statistical significance.

## 4. Discussion

### 4.1. Primary Findings

Our study showed that mothers of LGA neonates had significantly higher pre-pregnancy weight, height, and BMI. A moderate, but not high, CQI was associated with an increased risk of LGA neonates in the overall population across several adjusted models, though no clear linear trend was observed. Conversely, in women with GDM, a higher CQI was significantly associated with an increased risk of LGA in less adjusted models with a significant positive trend; however, this association became non-significant in the fully adjusted model. This likely reflects the confounding impact of maternal lifestyle or metabolic status, which may overshadow the independent role of carbohydrate quality in GDM pregnancies. When examining individual CQI components (glycemic index, dietary fiber, solid/total carbohydrate ratio, whole/total grain ratio) in the fully adjusted model, no significant associations with LGA risk were found in the total population or the GDM subgroup, although there was a non-significant trend towards increased LGA risk with higher dietary fiber intake in the GDM group. Notably, when considering carbohydrate quantity and quality combined, consuming ≥50% of energy from carbohydrates of low quality was significantly associated with a four-fold increase in the LGA risk of the total population. In the GDM subgroup, analyses of combined quality and quantity were hampered by limited data, yielding largely non-significant results with wide confidence intervals.

### 4.2. Interpretation of the Findings

The initial observation that mothers of LGA neonates in the total study population exhibited significantly higher pre-pregnancy weight and BMI is consistent with a substantial body of existing literature [27,28]. Elevated pre-pregnancy BMI is a well-established independent risk factor for LGA and macrosomia, with effects that are both independent of [29] and partly mediated through GDM status [30]. This association is largely attributed to the influence of maternal adiposity on insulin resistance and glucose metabolism, which can lead to increased nutrient transfer to the fetus and subsequent excessive growth [31]. These are confounding factors that are crucial to be adjusted for our investigated comparisons to be clinically relevant.

An interesting finding of our adjusted models was that a moderate CQI was associated with an increased risk of LGA compared to a low CQI in the overall population. We did not detect a clear linear trend as high CQI had no higher effect estimates compared to moderate CQI, suggesting a complex, potentially non-linear relationship that warrants further exploration. This may reflect heterogeneity in diet quality or unmeasured confounders within the moderate CQI group. However, the significant association between consuming ≥50% of energy from low-quality carbohydrates and an increased LGA risk in the total population aligns with the broader understanding that overall poor dietary quality, often characterized by refined carbohydrates and added sugars, contributes to metabolic dysregulation and adverse pregnancy outcomes which is supported by a meta-analysis on the matter [16]. Xue et al. reinforced this distinction, showing that poor-quality carbohydrates, particularly refined grains and added sugars, were associated with increased risk of excessive fetal growth and other pregnancy complications, independent of total intake [23]. This supports the idea that composite measures like CQI offer value, even if their relationship with LGA is less consistent than with GDM. It seems that CQI has not as clear an association with LGA as it does with GDM. A relevant cohort study depicted a more linear negative association between the consumption of higher CQI carbohydrates and the risk for GDM, reinforcing the preventive potential of carbohydrate quality before and during early pregnancy [32]. Taken together, these findings suggest that, while CQI has a clear inverse association with GDM, its effect on fetal growth and birthweight may be more complex. Nevertheless, high intake of low-quality carbohydrates appears to consistently increase the risk of excessive fetal growth.

In the subgroup of women with GDM, the initial significant association between a higher CQI and increased LGA risk in less adjusted models, which subsequently became non-significant in the most fully adjusted model, underscores the critical influence of confounding factors. Despite statistical non-significance in the fully adjusted models, the magnitude of some aORs, such as >6 for high CQI, may indicate a clinically meaningful trend that warrants confirmation in larger studies. GDM itself is a potent independent risk factor for LGA, and its presence can either mask or modify the effects of dietary quality. The attenuation of this association after comprehensive adjustment suggests that other variables, such as the severity of GDM, pre-pregnancy metabolic status, or other lifestyle factors, may be stronger determinants of LGA in this high-risk cohort. The non-significant trend towards increased LGA risk with higher dietary fiber intake in the GDM group is particularly noteworthy. This is consistent with findings from our earlier analysis of the same BORN 2020 cohort by Siargkas et al., in which we reported a counter-intuitive association between higher pre-pregnancy dietary fiber intake and increased LGA risk among women with GDM and normal pre-pregnancy BMI. That study also identified similar trends for vegetable protein intake, further emphasizing the complexity of nutritional influences on fetal overgrowth in this high-risk population [33]. This finding mirrors the non-linear and inconsistent associations we observed between CQI and LGA in the GDM subgroup. Additionally, a randomized controlled trial detected a positive association between low glycemic index and gestational weight in high-risk pregnancies for macrosomia [19]. Conversely, other evidence has shown no relationship between low glycemic index diets and LGA in GDM pregnancies [34]. These findings highlight the complex and context-dependent role of dietary fiber, which may vary depending on its type (e.g., soluble vs. insoluble), intake level, and the individual’s metabolic context. In addition, carbohydrate-related guidance in GDM should not be limited to quality alone. The amount of carbohydrate consumed, as well as the timing and distribution of intake across the day, may play a critical role in postprandial glucose control and fetal growth outcomes.

The absence of significant associations for individual CQI components (glycemic index, dietary fiber, solid/total carbohydrate ratio, whole/total grain ratio) with LGA risk in the fully adjusted models, across both the total population and the GDM subgroup, suggests that the composite CQI metric may be too broad to capture the nuanced effects of specific carbohydrate characteristics or that the individual effects are subtle and highly dependent on the overall dietary matrix. It is possible that individual components exert only modest effects on fetal growth or that CQI operates as a synergistic measure. Additionally, dietary recall limitations and underpowered subgroup analyses may have obscured associations. This aligns with the concept that dietary patterns, rather than isolated nutrients, often exert a more profound influence on health outcomes [35]. However, future research should also examine factors such as glycemic load, timing and structure of carbohydrate intake, and postprandial glycemic responses, which may offer more practical and clinically relevant targets for dietary counseling in GDM and pregnancy. Furthermore, the limitations encountered in analyzing combined carbohydrate quality and quantity in the GDM subgroup, resulting in largely non-significant findings with wide confidence intervals, are a common challenge in GDM research [32]. Such limitations often stem from smaller sample sizes or inherent difficulties in precise dietary assessment within this specific population, emphasizing the ongoing need for larger, well-powered studies to elucidate these complex relationships.

### 4.3. Strengths and Limitations

This study has several notable strengths. First, it is embedded within a well-defined longitudinal cohort (BORN 2020), allowing for detailed collection of dietary, anthropometric, and clinical data over time. The use of a validated food frequency questionnaire and a comprehensive CQI, encompassing multiple dimensions of carbohydrate intake (glycemic index, fiber density, grain type, and carbohydrate form), enhances the robustness of our dietary assessment. Additionally, outcome ascertainment relied on clinically verified birthweight data and gestational age, improving measurement accuracy. The use of multiple adjusted models accounting for key confounders such as pre-pregnancy BMI, gestational weight gain, and lifestyle factors provides a more reliable estimation of associations between carbohydrate quality and LGA risk.

However, certain limitations should be acknowledged. The observational nature of the study precludes causal inference. Despite adjusting for known confounders, residual confounding, particularly from unmeasured variables such as insulin sensitivity or dietary reporting bias, cannot be ruled out. Additionally, the use of a single regional cohort may limit generalizability to broader populations, particularly outside the Mediterranean dietary and cultural context. The findings related to the GDM subgroup were constrained by small sample size, leading to wide confidence intervals and reduced statistical power, especially in stratified analyses combining carbohydrate quality and quantity. This limited subgroup size, particularly among women with both GDM and LGA outcomes, may have hindered the detection of true associations and should be addressed in future studies with larger, more highly powered samples. Furthermore, the composite nature of the CQI may mask distinct effects of its individual components, as our results suggest limited associations when these were analyzed separately. Finally, dietary intake was assessed only at baseline, and changes in diet during pregnancy were not captured, potentially diluting true associations. Future studies with repeated dietary assessments and larger GDM-specific cohorts are warranted to clarify these complex interactions.

## 5. Conclusions

Our study adds to the limited evidence base on the role of carbohydrate quality in pregnancy outcomes. Increased intake of low-quality carbohydrates, characterized by high consumption of refined grains, added sugars, and low fiber, was significantly associated with a greater risk of delivering LGA infants in the general population. This highlights the detrimental impact of poor carbohydrate quality during pregnancy. Importantly, while the overall CQI did not exhibit a clear or linear association with LGA, our findings suggest that carbohydrate quality alone does not adequately capture the complexity of fetal overgrowth risk. In pregnancies complicated by GDM, high-quality carbohydrates were associated with a greater risk of LGA, probably indicating that total carbohydrate load, regardless of quality, should be carefully monitored. These findings support the need for nuanced dietary recommendations in pregnancy that consider both the quality and quantity of carbohydrate intake, particularly in high-risk groups such as women with GDM. Dietary counseling during pregnancy should emphasize reducing refined carbohydrates and promoting fiber-rich, high-quality carbohydrate sources to help prevent fetal overgrowth and related complications. However, even with multivariable adjustments, residual confounding, particularly from socioeconomic status, physical activity, or gestational weight gain, may persist. Future studies with larger sample sizes are needed to confirm these associations, especially in high-risk subgroups.

## Figures and Tables

**Table 1:** Maternal characteristics of the population by LGA and GDM status, N = 797.

Variable	Non-LGA N = 645	LGA N = 152	*p*-Value	GDM Non-LGA N = 94	GDM with LGA N = 23	*p* Value
Maternal age	32.41 (±4.97)	32.4 (±4.48)	0.98	34.39 (±4.72)	33.17 (±3.2)	0.24
Maternal age > 35	191 (29.61%)	46 (30.26%)	0.95	44 (46.81%)	7 (30.43%)	0.24
Weight	63 (57, 72)	66 (60, 77)	*p* < 0.001 ***	64.5 (58.25, 78)	68 (63.5, 80.5)	0.16
Height	165 (161, 170)	167 (162.75, 170)	0.006 **	164.7 (±6.28)	168.22 (±6.15)	0.017 *
BMI before pregnancy	22.7 (20.7, 25.8)	23.8 (21.6, 27.42)	0.004 **	23.55 (21.62, 28.43)	24.7 (22.65, 28.75)	0.45
BMI before pregnancy > 25	196 (30.39%)	64 (42.11%)	0.007 **	36 (38.3%)	10 (43.48%)	0.83
BMI before pregnancy > 30	76 (11.78%)	24 (15.79%)	0.23	21 (22.34%)	4 (17.39%)	0.81
Parity 0 1 2 3 4	339 (52.56%) 234 (36.28%) 62 (9.61%) 8 (1.24%) 2 (0.31%)	68 (44.74%) 63 (41.45%) 19 (12.5%) 2 (1.32%) 0 (0%)	0.1 0.27 0.36 1 -	52 (55.32%) 32 (34.04%) 9 (9.57%) 1 (1.06%)	8 (34.78%) 12 (52.17%) 3 (13.04%) 0 (0%)	0.13 0.17 0.91 -
Smoking	69 (10.7%)	12 (7.89%)	0.38	18 (19.15%)	3 (13.04%)	0.7
ART	49 (7.6%)	10 (6.58%)	0.8	9 (9.57%)	2 (8.7%)	0.1
Thyroid disease	89 (13.8%)	17 (11.18%)	0.47	9 (9.57%)	4 (17.39%)	0.48
GDM	94 (14.57%)	23 (15.13%)	0.96	94 (100%)	23 (100%)	-

LGA: large for gestational age; GDM: gestational diabetes mellitus; BMI: body mass index; ART: Assisted Reproductive Technologies; Thyroid disease: included hypothyroidism, Hashimoto’s thyroiditis, hyperthyroidism. Continuous variables are presented as mean ± standard deviation (SD) or median (interquartile range) depending on normality. Normality was assessed using the Shapiro–Wilk or Kolmogorov–Smirnov test as appropriate. Group comparisons were performed using the independent samples *t*-test for normally distributed variables, the Mann–Whitney U test for skewed distributions, and the Chi-squared or Fisher’s exact test for categorical variables. “*” denotes *p* < 0.05, “**” denotes *p* < 0.01, and “***” denotes *p* < 0.001.

**Table 2:** Carbohydrate Quality Index and association with LGA.

	CQI for LGA in Total Population					
Models	Low	Moderate		High		
		aOR (95% CI)	*p*-Value	aOR (95% CI)	*p*-Value	*p* for Trend
**Model 1**	**reference**	1.6 (1.03, 2.5)	0.037 *	1.29 (0.81, 2.07)	0.28	0.23
**Model 2**	**reference**	1.57 (1.01, 2.46)	0.046 *	1.26 (0.78, 2.02)	0.33	0.28
**Model 3**	**reference**	1.58 (1.01, 2.47)	0.044 *	1.26 (0.79, 2.03)	0.33	0.28
**Model 4**	**reference**	1.7 (1.08, 2.72)	0.023 *	1.44 (0.86, 2.4)	0.16	0.12
	**CQI for LGA in GDM**					
**Models**	**Low**	**Moderate**		**High**		***p* for Trend**
		**aOR (95% CI)**	***p*** **-Value**	**aOR (95% CI)**	***p*** **-Value**	
**Model 1**	**reference**	3.85 (0.72, 31.93)	0.15	6.74 (1.32, 56.66)	0.039 *	0.041 *
**Model 2**	**reference**	3.81 (0.71, 32.02)	0.15	6.64 (1.27, 57.48)	0.044 *	0.045 *
**Model 3**	**reference**	3.56 (0.65, 30.04)	0.18	6.27 (1.2, 54.33)	0.051	0.052
**Model 4**	**reference**	1.9 (0.28, 18.14)	0.53	3.05 (0.47, 30.22)	0.28	0.3

CQI: Carbohydrate Quality Index; LGA: Large for Gestational Age, GDM: Gestational Diabetes Mellitus; aOR: Adjusted Odds Ratio; CI: Confidence Interval; BMI: Body Mass Index; MD: Mediterranean Diet. Associations were estimated using multivariable logistic regression models. Adjustments were applied progressively: Model 1: adjusted for energy intake, maternal age, BMI before pregnancy, parity, physical activity, weight gain, thyroid disease, supplementation intake, sedentary hours per day; Model 2: model 1 plus smoking status; Model 3: model 2 plus alcohol intake; Model 4: model 3 plus adherence to MD by Trichopoulou. The reference category for CQI was the lowest tertile. “*” denotes *p* < 0.05.

**Table 3:** Adjusted associations between carbohydrate quality index and LGA risk.

Variable	Tertile/Group	LGA			GDM with LGA		
		aOR (95% CI)	*p*-Value	Power	aOR (95% CI)	*p*-Value	Power
Glycemic index (GI)	T2 (medium)	0.95 (0.61, 1.5)	0.85	0.135	0.76 (0.19, 2.76)	0.68	0.552
	T3 (high)	0.79 (0.49–1.28)	0.36	0.994	0.45 (0.07, 2.15)	0.34	1
Dietary fiber	T2 (medium)	1.38 (0.87, 2.22)	0.17	1	2.18 (0.43, 13.5)	0.36	1
	T3 (high)	1.25 (0.71, 2.2)	0.43	0.994	3.15 (0.59, 20.6)	0.19	1
Solid/Total carb ratio	T2 (medium)	0.98 (0.62, 1.55)	0.96	0.056	0.55 (0.13, 2.16)	0.4	0.994
	T3 (high)	1.24 (0.79, 1.95)	0.34	0.992	0.66 (0.16, 2.48)	0.55	0.87
Whole/Total grain ratio	T2 (medium)	1.31 (0.83, 2.03)	0.23	1	2.3 (0.49, 12.28)	0.3	1
	T3 (high)	1.21 (0.76, 1.91)	0.41	0.969	2.29 (0.55, 11.38)	0.27	1

CQI: Carbohydrate Quality Index; LGA: Large for Gestational Age. Associations were derived using multivariable logistic regression (Model 4), adjusted for energy intake, maternal age, BMI before pregnancy, parity, physical activity, gestational weight gain, thyroid disease, supplementation intake, sedentary hours per day, smoking status, alcohol intake, and adherence to the Mediterranean Diet (Trichopoulou score). The lowest tertile (T1) served as the reference group for each CQI component. Statistical power was calculated post hoc for each model.

**Table 4:** Carbohydrate quality and quantity in relation to LGA outcome.

	Carbohydrate Quality Index in LGA		
	Low	Moderate	High
**Carbohydrate Intake (% E)**			
≤40%	reference	reference	reference
40–50%	1.78 (0.83, 3.82), *p* = 0.14	1.18 (0.57, 2.46), *p* = 0.65	0.87 (0.4, 1.88), *p* = 0.73
≥50%	4.25 (1.53, 11.67), *p* = 0.005 *	1.63 (0.69, 3.81), *p* = 0.25	0.53 (0.16, 1.52), *p* = 0.26
	**Carbohydrate Quality Index in GDM with LGA**		
**Carbohydrate Intake (% E)**			
≤40%	reference	reference	reference
40–50%	6.14 × 10^−10^ (-,-), *p* = 0.1	- (-,-), *p* = 0.1	1.12 (0.11, 10.47), *p* = 0.92
≥50%	0.01 (-,-), *p* = 0.1	- (-,-), *p* = 0.1	4.05 (0.13, 165.61), *p* = 0.43

LGA: large for gestational age; GDM: Gestational Diabetes Mellitus; %E: Percent of total energy intake. Adjusted odds ratios were estimated using multivariable logistic regression (Model 4), controlling for energy intake, maternal age, pre-pregnancy BMI, parity, physical activity, gestational weight gain, thyroid disease, supplementation use, sedentary behavior, smoking status, alcohol intake, and adherence to the Mediterranean Diet (Trichopoulou score). The lowest carbohydrate intake group (≤40% of energy) within each Carbohydrate Quality Index category served as the reference. “-” indicates insufficient data for reliable estimation in that subgroup. “*” denotes *p* < 0.05.

## Data Availability

Data are not publicly available due to privacy restrictions.

## References

[1] Hong Y.H., Lee J.-E. (2021). Large for gestational age and obesity-related comorbidities. J. Obes. Metab. Syndr..

[2] Ahlsson F., Gustafsson J., Tuvemo T., Lundgren M. (2007). Females born large for gestational age have a doubled risk of giving birth to large for gestational age infants. Acta Paediatr..

[3] Berntorp K., Anderberg E., Claesson R., Ignell C., Källén K. (2015). The relative importance of maternal body mass index and glucose levels for prediction of large-for-gestational-age births. BMC Pregnancy Childbirth.

[4] Luoto R., Kinnunen T.I., Aittasalo M., Kolu P., Raitanen J., Ojala K., Mansikkamäki K., Lamberg S., Vasankari T., Komulainen T. (2011). Primary prevention of gestational diabetes mellitus and large-for-gestational-age newborns by lifestyle counseling: A cluster-randomized controlled trial. PLoS Med..

[5] Chawla R., Badon S.E., Rangarajan J., Reisetter A.C., Armstrong L.L., Lowe L.P., Urbanek M., Metzger B.E., Hayes M.G., Scholtens D.M. (2014). Genetic risk score for prediction of newborn adiposity and large-for-gestational-age birth. J. Clin. Endocrinol. Metab..

[6] Boubred F., Pauly V., Romain F., Fond G., Boyer L. (2020). The role of neighbourhood socioeconomic status in large for gestational age. PLoS ONE.

[7] Coustan D.R., Lowe L.P., Metzger B.E., Dyer A.R. (2010). The Hyperglycemia and Adverse Pregnancy Outcome (HAPO) study: Paving the way for new diagnostic criteria for gestational diabetes mellitus. Am. J. Obstet. Gynecol..

[8] Azoulay L., Bouvattier C., Christin-Maitre S. (2022). Impact of intra-uterine life on future health. Annales d’Endocrinologie.

[9] Gluckman P.D., Cutfield W., Hofman P., Hanson M.A. (2005). The fetal, neonatal, and infant environments—The long-term consequences for disease risk. Early Hum. Dev..

[10] Sakuyama H., Katoh M., Wakabayashi H., Zulli A., Kruzliak P., Uehara Y. (2016). Influence of gestational salt restriction in fetal growth and in development of diseases in adulthood. J. Biomed. Sci..

[11] Öztürk H.N.O., Türker P.F. (2021). Fetal programming: Could intrauterin life affect health status in adulthood?. Obstet. Gynecol. Sci..

[12] Danley S., Laban D. (2025). The Influence of Maternal Nutrition on Fetal Development and Birth Outcomes. World Sci. News.

[13] Dhobale M. (2017). Neurotrophic factors and maternal nutrition during pregnancy. Vitam. Horm..

[14] Sweeting A., Mijatovic J., Brinkworth G.D., Markovic T.P., Ross G.P., Brand-Miller J., Hernandez T.L. (2021). The carbohydrate threshold in pregnancy and gestational diabetes: How low can we go?. Nutrients.

[15] Filardi T., Panimolle F., Crescioli C., Lenzi A., Morano S. (2019). Gestational diabetes mellitus: The impact of carbohydrate quality in diet. Nutrients.

[16] Zhang R., Han S., Chen G.-C., Li Z.-N., Silva-Zolezzi I., Parés G.V., Wang Y., Qin L.-Q. (2018). Effects of low-glycemic-index diets in pregnancy on maternal and newborn outcomes in pregnant women: A meta-analysis of randomized controlled trials. Eur. J. Nutr..

[17] Moses R.G., Casey S.A., Quinn E.G., Cleary J.M., Tapsell L.C., Milosavljevic M., Petocz P., Brand-Miller J.C. (2014). Pregnancy and Glycemic Index Outcomes study: Effects of low glycemic index compared with conventional dietary advice on selected pregnancy outcomes. Am. J. Clin. Nutr..

[18] Louie J.C.Y., Markovic T.P., Perera N., Foote D., Petocz P., Ross G.P., Brand-Miller J.C. (2011). A randomized controlled trial investigating the effects of a low–glycemic index diet on pregnancy outcomes in gestational diabetes mellitus. Diabetes Care.

[19] Walsh J.M., McGowan C.A., Mahony R., Foley M.E., McAuliffe F.M. (2012). Low glycaemic index diet in pregnancy to prevent macrosomia (ROLO study): Randomised control trial. BMJ.

[20] Liu L., Liu Z., Duan B., Zhang Q., Zhou Z., Liu W. (2023). Effects of a low glycemic index or low glycemic load diet on pregnant women at high risk of gestational diabetes: A meta-analysis of randomized controlled trials. Nutr. Metab. Cardiovasc. Dis..

[21] Schulz R., Slavin J. (2021). Perspective: Defining carbohydrate quality for human health and environmental sustainability. Adv. Nutr..

[22] Maghoul A., Khonsari N.M., Asadi S., Abdar Z.E., Ejtahed H.-S., Qorbani M. (2023). Dietary carbohydrate quality index and cardio-metabolic risk factors. Int. J. Vitam. Nutr. Res..

[23] Xue L., Chen X., Sun J., Fan M., Qian H., Li Y., Wang L. (2024). Maternal dietary carbohydrate and pregnancy outcomes: Quality over quantity. Nutrients.

[24] Apostolopoulou A., Tranidou A., Tsakiridis I., Magriplis E., Dagklis T., Chourdakis M. (2024). Effects of Nutrition on Maternal Health, Fetal Development, and Perinatal Outcomes. Nutrients.

[25] Apostolopoulou A., Magriplis E., Tsekitsidi E., Oikonomidou A.C., Papaefstathiou E., Tsakiridis I., Dagklis T., Chourdakis M. (2021). Development and validation of a short culture-specific food frequency questionnaire for Greek pregnant women and their adherence to the Mediterranean diet. Nutrition.

[26] Tsagkari A., Pateras K., Ladopoulou D., Kornarou E., Vlachadis N. (2020). Birthweight by gestational age reference centile charts for Greek neonates. medRxiv.

[27] Mao K., Gao Y., Li S., Chi L. (2024). A retrospective cohort study on the influencing factors for macrosomia in singleton pregnancies. Medicine.

[28] Jenabi E., Salehi A.M., Farashi S., Salimi Z. (2024). The environmental risk factors associated with fetal macrosomia: An umbrella review. Pediatr. Neonatol..

[29] Alfadhli E.M. (2021). Maternal obesity influences birth weight more than gestational diabetes. BMC Pregnancy Childbirth.

[30] Song X., Shu J., Zhang S., Chen L., Diao J., Li J., Li Y., Wei J., Liu Y., Sun M. (2022). Pre-pregnancy body mass index and risk of macrosomia and large for gestational age births with gestational diabetes mellitus as a mediator: A prospective cohort study in Central China. Nutrients.

[31] Group H.S.C.R. (2010). Hyperglycaemia and Adverse Pregnancy Outcome (HAPO) Study: Associations with maternal body mass index. BJOG Int. J. Obstet. Gynaecol..

[32] Fernández-González E., Martínez-González M.Á., Bes-Rastrollo M., Suescun-Elizalde D., Basterra-Gortari F.J., Santiago S., Gea A. (2023). Association between pre-conceptional carbohydrate quality index and the incidence of gestational diabetes: The SUN cohort study. Br. J. Nutr..

[33] Siargkas A., Tranidou A., Magriplis E., Tsakiridis I., Apostolopoulou A., Xenidis T., Pazaras N., Chourdakis M., Dagklis T. (2025). Impact of Maternal Macronutrient Intake on Large for Gestational Age Neonates’ Risk Among Women with Gestational Diabetes Mellitus: Results from the Greek BORN2020 Cohort. Nutrients.

[34] Louie J.C.Y., Markovic T.P., Ross G.P., Foote D., Brand-Miller J.C. (2015). Effect of a low glycaemic index diet in gestational diabetes mellitus on post-natal outcomes after 3 months of birth: A pilot follow-up study. Matern. Child Nutr..

[35] Pittyanont S., Suriya N., Sirilert S., Tongsong T. (2024). Comparisons of the Rates of Large-for-Gestational-Age Newborns between Women with Diet-Controlled Gestational Diabetes Mellitus and Those with Non-Gestational Diabetes Mellitus. Clin. Pract..

